# Apoptosis-related gene expression induced by Colombian propolis samples in canine osteosarcoma cell line

**DOI:** 10.14202/vetworld.2021.964-971

**Published:** 2021-04-22

**Authors:** Dolly Patricia Pardo-Mora, Oscar Julián Murillo, Mauricio Rey-Buitrago, Mónica Losada-Barragán, Jaime Fabian Cruz Uribe, Karina Basso Santiago, Bruno José Conti, Eliza de Oliveira Cardoso, Fernanda Lopes Conte, Rafael María Gutiérrez, Orlando Torres García, José Maurício Sforcin

**Affiliations:** 1 Department of Animal Health, Facultad de Medicina Veterinaria, Universidad Antonio Nariño, Bogotá, Colombia; 2 Department of Morphology, Facultad de Medicina, Universidad Nacional de Colombia, Bogotá, Colombia; 3 Department of Chemical and Biological Sciences, São Paulo State University (UNESP), Institute of Biosciences, Campus Botucatu, Brazil

**Keywords:** apoptosis, cytotoxicity, osteosarcoma, propolis

## Abstract

**Background and Aim:**

Osteosarcoma (OSA) is the most common bone tumor in canines and humans. This study aimed to assess the cytotoxic and apoptotic effects of Colombian propolis samples on a canine OSA cell line (OSCA-8) by evaluating the expression of *BCL-2*, *BAX*, *CASPASE 9*, *CASPASE 8*, and *TNFR1* genes involved in the apoptosis pathway.

**Materials and Methods:**

After treating the cells with five Colombian propolis samples (Usm, Met, Fus, Sil, and Caj), we evaluated cell viability and lactate dehydrogenase (LDH) release. Early and late apoptosis was determined by flow cytometry using annexin V/propidium iodide. Furthermore, the effects of three selected samples on gene expression were analyzed by real-time polymerase chain reaction.

**Results:**

The Colombian propolis samples reduced OSCA-8 cell viability and increased LDH release. All samples induced apoptosis significantly and upregulated *BCL-2* and *CASPASE 8* expression. Usm and Sil increased *BAX* expression, Met and Sil induced *CASPASE 9* expression, and Usm increased *TNFR1*.

**Conclusion:**

Colombian propolis samples exhibited cytotoxic and apoptotic effects on canine OSA cells, and *CASPASE 8* upregulation indicated apoptosis induction by the extrinsic pathway.

## Introduction

Osteosarcoma (OSA) is one of the most common cancer types in adolescents but diagnosed less frequently than other neoplasms [1,2]. OSA is also the leading bone tumor in canines apart from humans, with a higher prevalence in canines; however, both species have a relatively poor prognosis. These species have similar morphological and physiological characteristics and gene expression; thus, the canine OSA is a suitable model to study this disease in humans [1,3]. Moreover, considering the development of multidrug resistance to chemotherapy [3], resistance mechanisms and gene expression can be evaluated using canine OSA cell lines for predicting drug response [4]. Hence, animal models can be used to investigate novel treatment strategies to improve the therapeutic response in humans.

Apoptosis is a programmed cell death that maintains a healthy survival/death balance [5]. Inducing apoptosis is useful for treating OSA and other neoplasms, and the apoptotic pathways may be regulated at several levels [3]. Apoptosis may be triggered by extrinsic and intrinsic pathways; the former is activated by specific ligands in death receptors present in the cell membrane, whereas the latter involves proapoptotic protein release from the mitochondria [2]. Tumor necrosis factor (TNF) receptors such as TNFR1 and FAS may induce the extrinsic pathway. After ligation of these receptors, several intracellular proteins, including certain procaspases, are recruited to the receptors’ cytosolic domains, forming a death-inducing signaling structure that activates CASPASES 8 and 10 [5,6]. Meanwhile, the intrinsic pathway is regulated by BCL-2 proteins such as BCL-2 and BAX [7], and the BCL-2/BAX ratio can indicate cell apoptosis or survival [8,9]. Therefore, these pathways may serve as targets to develop new drugs with antitumor effects [7].

Propolis is a resinous material produced by bees from different botanical sources worldwide. Propolis displays numerous pharmacological properties, including its cytotoxic action *in vitro* and antitumor activity *in vivo*. In Colombia, propolis samples collected in different regions have been chemically characterized, presenting similar components to those found in samples from the *Clusia* plant, which is native in other tropical countries, such as Cuba and Venezuela [10]. However, Colombian propolis contains diterpenes and triterpenes, which are still currently poorly known, and components such as eicosyl coumarate and garcinoic acid, which are not observed in other propolis types [11,12]. Propolis may affect both the intrinsic and extrinsic apoptosis pathways [13,14]. In human breast cell line MCF-7, propolis increases the activity of CASPASE 3 [15,16] and the immunoreactivity of CASPASES 6, 8, and 9 in cancer cells [13]; in human leukemic cell line U937, it decreases *BCL-2* expression [14,15]. According to a previous study focusing on potential cytotoxicity, Colombian propolis samples can induce apoptosis in canine OSA cells [11].

However, the effects of these samples on the expression of genes involved in apoptosis pathways remain unexplored. Therefore, this study aimed to evaluate the effects of different Colombian propolis extracts on the viability, lactate dehydrogenase (LDH) leakage, and apoptosis induction in canine OSA cells, particularly OSCA-8, by analyzing the expression of *BAX, BCL-2, CASPASE 9, CASPASE 8*, and *TNFR1* genes.

## Materials and Methods

### Ethical approval

This research was approved by the Ethics Committee of Antonio Nariño University, Colombia (act 004 - 2016).

### Study period and location

Samples were collected from July to December 2015 and the study was performed from February to June 2018, in the Universidad Antonio Nariño, Colombia.

### Propolis ethanolic extracts

We collected five propolis samples from different regions of Colombia, as follows: Usme, Bogotá District Capital (Usm, 4°29′05′′N 74°07′44′′W); Puerto Lopez, Meta (Met, 4°01′47′′N 72°41′50′′W); Fusagasuga, Cundinamarca (Fus, 4°19′15′′N 74°24′08′′W); Silvia, Cauca (Sil, 2°37′32′′N 76°23′29′′W); and Cajibio, Cauca (Caj, 2°39′15′′N 76°39′34′′W). From July to December of 2015, the samples were collected using plastic nets and by scraping the hives. Moreover, they were characterized chemically [11]. After grinding all the samples, we dissolved 4.5 g of propolis in 15 mL of 70% ethanol. The extracts were kept under moderate shaking, with no bright light, and at 20ºC. After 24 h, we filtered the extracts and calculated the dry weight (mg/mL) of each extract.

### Cell line maintenance

OSCA-8 cells (Kerafast, Inc., Boston, MA, USA) were maintained in Dulbecco’s Modified Eagle’s Medium (Santa Cruz Biotechnology Inc., USA) supplemented with 10% fetal calf serum and antibiotic-antimycotic solution (100×, Gibco, Thermo Fisher Scientific Inc., USA). In addition, cells were maintained at 37°C in a humidified 5% CO_2_ atmosphere.

### Cell viability determination by 3-(4,5-dimethylthiazol-2-yl)-2,5-diphenyltetrazolium bromide (MTT) assay

We cultured OSCA-8 cells (3-4 passages) in 96-well plates and placed 100 μL (1×10^5^ cells/mL) in each well. These cells were then incubated overnight for adherence and exposed to propolis extracts (10, 25, 50, and 100 μg/mL) for 24, 48, and 72 h. Next, we evaluated the effect of 70% ethanol (propolis solvent) on cell viability, with its concentration identical to the highest propolis concentration (0.48%). We also evaluated formazan formation using Cytoselect™ MTT Cell Proliferation Assay (Cell Biolabs, Inc., USA) and by measuring the absorbance in an automated plate reader (Multiskan FC, Thermo Scientific Inc) at 540 nm. We considered the absorbance of untreated cells as 100% cell viability. Assays were performed twice in quadruplicate for each propolis sample.

### LDH leakage assay

We cultured OSCA-8 cells in 96-well plates and added 150 mL of the study sample to each well. Next, the cells (1×10^5^ cells/mL) were incubated overnight for adherence and then incubated with propolis samples (10, 25, 50, and 100 μg/mL) for 48 and 72 h. In evaluating LDH release, we measured the absorbance in an automated plate reader (Multiskan FC, Thermo Scientific Inc.) at 450 nm. For the positive control (Triton X-100 Solution) included in the Cytoselect™ LDH Cytotoxicity Assay Kit (Cell Biolabs Inc.), the absorbance was considered as 100% cytotoxicity. We conducted the experiments twice in quadruplicate for each extract.

### Annexin V/propidium iodide (PI) flow cytometric analysis

The apoptotic effects of propolis on OSCA-8 cells were determined using Annexin V Apoptosis Kit (Santa Cruz Biotechnology Inc.). For 48 and 72 h, a 24-well plate containing 1×10^5^/mL canine OSA cells was exposed to five propolis extracts at 50 mg/mL. This concentration and the time period were selected according to our previous findings. On the basis of the MTT assay, this concentration was near the inhibitory concentration (IC) 50% (IC_50_). The cells were then harvested, centrifuged, and washed with phosphate-buffered saline (1×). For annexin binding, we used the buffered solution subsequently added with 0.5 μg of annexin V and 0.35 mg of PI. Thereafter, we incubated the cells for 15 min at 20ºCand analyzed using the flow cytometer BD CSampler™ BD Accuri. This tool recorded at least 20,000 events for each analysis, performed independently at least thrice. The data were analyzed using the CFlow Plus software (BD Biosciences, USA).

### RNA extraction, cDNA synthesis, and quantitative real-time polymerase chain reaction (qPCR)

For molecular assays, we selected Usm, Met, and Sil samples according to cytotoxic effect and chemical composition. Fus exerted a cytotoxic effect (LDH assay) only at 100 μg/mL, and Caj had a chemical composition similar to Sil; thus, they were not chosen. Canine OSA cells (1×10^5^/mL) were then treated with the three selected propolis extracts at 25 and 50 μg/mL; these concentrations were near IC_25_ according to the cell viability and cytotoxicity results. The expression of *BAX*, *BCL-2*, *CASPASE 9*, *CASPASE 8*, and *TNFR1* in propolis-treated OSA cells was compared with those in untreated ones. Doxorubicin (0.5 μM) was used as a positive control, and each experiment was performed in triplicate.

After 48 h of treatment, the cells were harvested for total RNA extraction using a commercial kit (InviTrap^®^ Spin Universal RNA Mini Kit [Molecular Stratec, Germany]) according to the manufacturer’s protocol. RNA was qualitatively evaluated by electrophoresis in 2% agarose gel and spectrophotometrically quantitated in a Nanodrop 2000 spectrophotometer (Thermo Scientific). The index purity (A260/A280) of all RNA samples was >2.10. Meanwhile, we synthesized cDNA using the ProtoScript® M-MuLV First Strand cDNA Synthesis Kit (Biolabs, Inc.). Finally, 400 ng of total RNA was reverse transcribed into cDNA to obtain cDNA at 8 ng/μL.

The qPCR analysis was performed using 2 mL of cDNA. Primers were forwarded and reversed at 500 nM, and 5 mL of FastStart Essential DNA Green Master 2× (Roche, USA) was used, obtaining a final volume of 10 μL. All of these data were analyzed thrice using LightCycler^®^ 96 (Roche). The oligonucleotide primers were sequenced using the BLAST program of the National Centre for Biotechnology Information (http://www.ncbi.nlm.nih.gov/blast), in accordance with the previous studies [17] and then synthesized by Macrogen^®^ (Korea). Table-1 lists the primer sequences.

**Table 1:** Sequences of the primers for qPCR.

	Primers sequence forward (5’-3’)	Primers sequence reverse (5’-3’)	Product length (bp)
*B2M*	GTTTCCTGGCCTTGCTCCTC	ACCCTGACACGTAGCAGTTC	158
*BAX*	GTTGCAGAGGATGATCGCAG	TGATGGTCCTGATCAGCTCGG	185
*BCL-2*	GGGGTCATGTGTGTGGAGAG	CAAACAGAGGCTGCATGGTG	169
*CASPASE 9*	GGGAAGCCCAAGCTCTTCTT	AGTGGGCAAACTAGACACGG	183
*CASPASE 8*	GATGCGGATGCGTTGAGTAA	AGCCATAGATGATGCCCTTGT	181
*TNFR1*	GCCTCTTTGGTATTTGCGTTT	CTCTTTCGTCGGTGTTGGTTT	113

The reaction conditions were 95°C for 300 s, 45 cycles of 95°C for 10 s, and 56-57°C for 30 s, followed by melting curve analysis at 95°C for 10 s, 65°C for 60 s, and 97°C for 1 s. The expression of the target genes was normalized using B*2-MICROGLOBULIN* (*B2M*) as an endogenous control because it is highly stable and has been recommended previously [18]. Negative controls without a cDNA template were included for each gene. Melting curves and 2% agarose gel electrophoresis were employed to confirm qPCR specificity.

### Statistical analysis

Data are expressed as means and standard deviation using GraphPad Prism 5 software (http://www.graphpad.com/quickcalcs/confInterval1.cfm). Differences between treatments and control groups were determined by one-way and two-way analysis of variance followed by Newman–Keuls multiple comparison tests and Bonferroni tests, respectively. Differences were considered statistically significant at p<0.05.

The qPCR results were analyzed using the 2^−∆∆Ct^ method, and the different expression levels were calculated as fold change [19]. Through this method, the relative quantification was determined by comparing the normalized Ct values (∆∆Ct) between treated and untreated samples. The assays were performed in duplicate in three independent experiments. Statistical differences between *BAX*, *BCL-2*, *CASPASE 9*, *CASPASE 8*, and *TNFR1* expression levels from exposed and non-exposed tumor cells to propolis extracts were identified using the Kruskal–Wallis test.

## Results

### Cell viability

**Figure-1:**
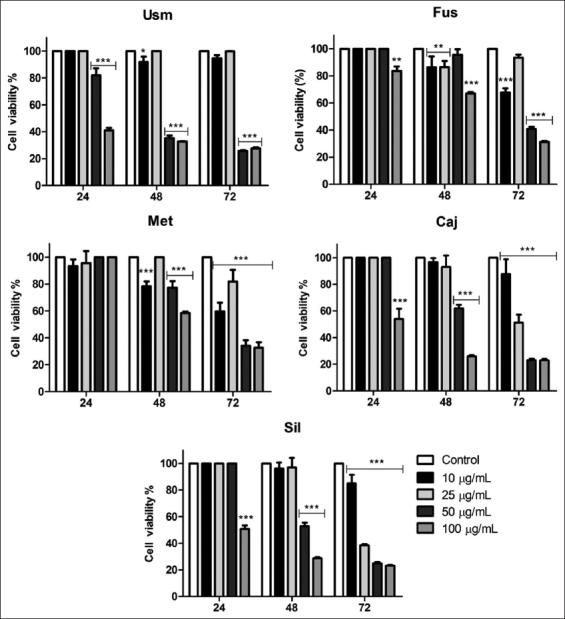
Percentage (%) of OSCA-8 cell viability after incubation with different propolis samples collected in Colombia (Usm, Fus, Met, Caj, and Sil) after 24, 48, and 72 h (mean±SD). *p<0.05; **p<0.01; ***p<0.001 versus control.

After 24 h of incubation, Usm at 50 and 100 μg/mL exerted a cytotoxic effect; however, Fus, Caj, and Sil only affected OSCA-8 cell viability at 100 μg/mL, while Met did not. After 48 h, Caj and Sil at 50 and 100 μg/mL exhibited an inhibitory effect, while Usm, Fus, and Met were efficient at 10, 50, and 100 μg/mL. After 72 h, the highest effect of all samples on cellular viability occurred: Usm (50 and 100 μg/mL), Fus (10, 50, and 100 μg/mL), and Met, Caj, and Sil (all concentrations) (Figure-1).

The effect of 70% ethanol (propolis solvent) on cell viability was not significant. Meanwhile, doxorubicin (4 μM) reduced cell viability to 62.06%±3.61% and 46.85%±6.85% after 48 and 72 h, respectively (data not shown).

### LDH release by OSCA-8 cells

**Figure-2:**
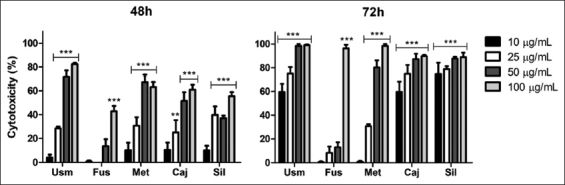
Percentage (%) of OSCA-8 cell cytotoxicity determined by lactate dehydrogenase release after incubation with five propolis samples collected in Colombia (Usm, Fus, Met, Caj, and Sil) for 48 and 72 h (mean±SD). **p<0.01; ***p<0.001 versus control.

Propolis samples exerted a cytotoxic action in a time-concentration-dependent manner. Cytotoxicity was lowest in Fus. Meanwhile, Usm, Met, Caj, and Sil presented >50% of cytotoxicity, with concentrations of >10 μg/mL after 72 h (Figure-2). In doxorubicin (4 μM), cytotoxicity was 37.45% and 88.91% at 48 and 72 h, respectively (data not shown).

### Apoptosis induction

**Figure-3:**
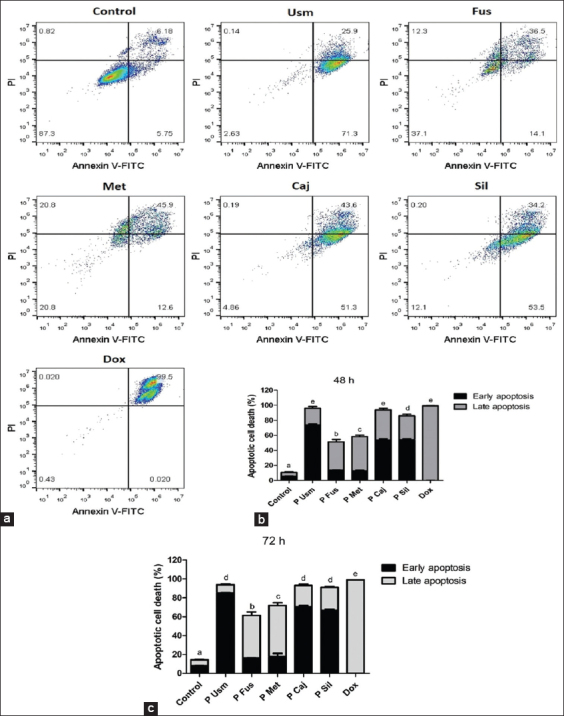
Apoptosis induction by Colombian propolis samples in OSCA-8 cells after 48 h of treatment was determined by flow cytometry. (a) Dot plot indicating the percentages in each panel: Early apoptotic (lower right quadrant), late apoptotic (upper right quadrant), and necrotic (upper left quadrant) cells. (b and c) Graph bars represent the percentage of cells in early and late apoptotic stages (mean±SD) after 48 h and 72 h. Different letters indicate significant differences (p<0.05).

Propolis extracts markedly increased OSCA-8 cell apoptosis. The total apoptosis in untreated cells was 10.77±2.22%, whereas that in treated cells ranged from 51.43±6.05% to 95.97±2.14%. Apoptotic effects were highest on OSCA-8 cells treated with Usm, Caj, and Sil. Similarly, doxorubicin induced apoptosis by >99% (Figure-3). The rate of early/late apoptosis and the total apoptosis increased after 72 h of incubation with propolis samples (Figure-3).

### *BAX, BCL-2, CASPASE 9, CASPASE 8*, and *TNFR1* gene expression

The 2^−∆∆Ct^ method revealed that *BAX* expression increased non-significantly in cells exposed to Usm (2.36±1.14) and Sil (1.89±0.25) compared with that in the control. Although the differences were not statistically significant, propolis treatments affected the expression level of several genes biologically. *BCL-2* expression also increased in propolis-treated cells but decreased in doxorubicin-treated cells. *CASPASE 9* expression was upregulated after treatment with propolis from Met (1.80±0.60) and Sil (1.79±0.68). The *BCL-2/BAX* ratio was >1 in cells treated with all propolis samples; thus, *BCL-2* has a possible antiapoptotic effect on the intrinsic pathway.

**Figure-4:**
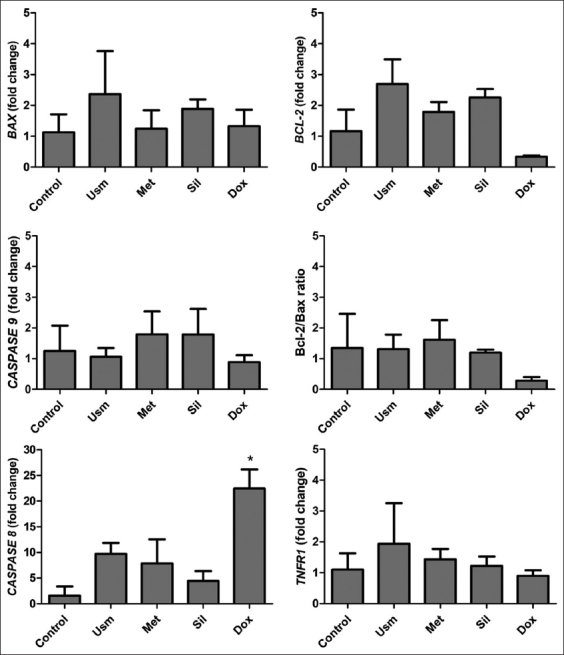
Propolis effects on *BAX, BCL-2, CASPASE 9, CASPASE 8*, and *TNFR1* gene expression and on the ratio *BCL-2/BAX*. Doxorubicin (1 μM) was used as a positive control (mean±SD). *p<0.05.

Propolis from Usm, Met, and Sil increased *CASPASE 8* expression by 9.70-±1.77-, 7.87-±3.83-, and 4.44-±1.56-fold, respectively. Only the propolis from Usm upregulated *TNFR1* to 1.94-±1.08-fold. Doxorubicin (0.5 mM) also increased *CASPASE 8* expression significantly (p<0.05) but downregulated *BCL-2* non-significantly (Figure-4).

## Discussion

This study investigated the potential cytotoxic activity of Colombian propolis samples on canine OSA cells by incubating such cells with different concentrations of propolis collected in different Colombian regions. As observed by the enzymatic conversion of MTT into formazan in the mitochondria, propolis samples reduced OSCA-8 cell viability. Overall, all the samples were similar in terms of decreasing OSCA-8 cell viability, without being influenced by the propolis solvent (70% ethanol). This finding is consistent with the previous studies, suggesting that propolis action is exclusively caused by its constituents [20,21]. In addition, propolis samples induced a cytotoxic effect toward canine OSA cells, correlated with the LDH leakage into the culture medium after cell membrane rupture. Used as a positive control, doxorubicin decreased cell viability, as expected, and induced apoptosis [22,23].

Propolis biological effects result from a synergistic action of its components [24]. Investigating the chemical composition of a given sample and its geographical origin is also necessary to ensure data reproducibility and the study’s scientific value [10]. Therefore, the present study used propolis samples characterized previously. Propolis from Usm contained approximately 37% of diterpenes and triterpenes, which are both insufficiently identified, 2.2% of flavonoids (kaempferol), and 3.4% of eicosyl coumarate. Fus and Met propolis samples contained diterpenes, benzophenones, triterpenes, and >22% of unknown compounds. Met propolis had 22% of nemorosone. Caj and Sil propolis samples had the highest amount of triterpenes (49.6% and 51.4%, respectively). In addition, Sil contained 7.9% of flavonoids [11].

In the present study, Colombian propolis ­samples induced apoptosis in OSCA-8 cells. Early and late apoptosis was determined using Annexin V/IP. All Colombian propolis extracts significantly increased the total apoptosis. The propolis samples Usm, Met, Caj, and Sil induced the highest number of cells undergoing apoptosis. Colombian propolis yielded higher total apoptosis than other propolis types with the same concentration as found in other studies [15,20,25]. Considering that the chemical composition of the propolis samples was different, the biological activities of eicosyl coumarate, diterpenes, triterpenes, and flavonoids present in the Usm, Met, Caj, and Sil propolis types require further investigation, especially because some components were different from those reported for propolis from other regions worldwide.

In addition, the propolis samples upregulated the genes *BAX, BCL-2, CASPASE 8* and *9*, and *TNFR1* involved in the apoptosis. Data related to gene expression were normalized to *B2M*, which was used as an endogenous control. Although the differences were not statistically significant, propolis treatments biologically affected the expression levels of such genes. Genes involved in the intrinsic pathway such as *BAX* were upregulated in cells exposed to Usm and Sil, as well as *CASPASE 9* in cells treated with Met and Sil. However, *BCL-2*, which presents an anti-apoptotic activity, was also upregulated in the cells treated with all propolis samples. The expression levels of *BAX* and *BCL-2* are similar to those in human bladder carcinoma cells exposed to Brazilian red propolis [8]. Sulaiman *et al*. [14] observed that in human leukemia cells treated with propolis from Iraq, BAX expression increased, but the BCL-2 expression decreased. Conversely, Japanese propolis did not change BAX expression but decreased BCL-2 expression [15]. *CASPASE 9* was upregulated in a bladder carcinoma cell line exposed to 50 μg/mL concentration of Brazilian red propolis, but it downregulated when 100 μg/mL was used, suggesting that propolis could activate independent caspase apoptosis pathways [8]. Mexican propolis also induced CASPASE 3 and 9 activation in lymphoma cells [20].

Chrysin is a flavone present in some propolis types; it increases the apoptosis induced by TRAIL and activates the CASPASE 8 in human cell lines [26]. In chondrosarcoma cells, curcumin, which is a bioactive component isolated from *Curcuma longa*, upregulated *FAS* and *FASL* and increased *CASPASE 3* and *8* activities [27]. These findings are similar to the action of Colombian propolis samples, that is, increased *CASPASE 8* expression. *TNFR1* participates in the extrinsic apoptosis pathway, but only Usm propolis increased its expression level in canine OSA cells. The Usm propolis contains eicosyl coumarate, which is an ester that can induce apoptosis selectively in cancer cells [28], and kaempferol, which exerts apoptotic effects on human OSA cells [29]. These two components were present in the Usm propolis sample and can be associated with the apoptotic effect observed in our study.

The propolis Sil mainly contained triterpenes and flavonoid aglycones. Both chemical groups exhibit anticancer activities [30,31] and may be involved in the apoptotic effect of *BAX*, *CASPASE 8*, and *CASPASE 9* expression in canine OSA cells. Meanwhile, the propolis Met mainly stimulated the *CASPASE 8* gene expression, and its activity may be associated with a high benzophenone (mainly nemorosone) content. Nemorosone displays a cytotoxic activity against cancer cell lines [32,33]. Furthermore, doxorubicin led to the highest level of total apoptosis, the lowest *BCL-2/BAX* ratio, and the highest *CASPASE 8* expression level, similar to the results of Sharifi *et al*. [23].

Finally, other components found in Colombian propolis should be isolated, and their cytotoxic effect on tumor cells should be evaluated. The action of Colombian propolis samples in other genes involved in apoptosis extrinsic and extrinsic pathways also require investigation.

## Conclusion

Colombian propolis samples reduced the viability of OSCA-8 cells, inducing cytotoxic effects and consequently, LDH release. In addition, these propolis samples induced apoptosis and apoptosis-related gene expression.

## Authors’ Contributions

DPP, RMG, and OTG: Conceptualization. DPP: Methodology and laboratory experiments. DPP, MR, ML, JFCU, KBS, BJC, EOC, OJM, FLC, RMG, OTG, and JMS: Interpretation of the data. DPPM: Manuscript writing. All authors read and approved the final manuscript.
